# Developing accessible and affordable tests to support mpox immunosurveillance and vaccine studies

**DOI:** 10.1080/22221751.2025.2576581

**Published:** 2025-10-16

**Authors:** Sian E. Faustini, Scott Jones, Christopher A. Green, Ashley D. Otter, Alex G. Richter, Jennifer L. J. Heaney

**Affiliations:** a Clinical Immunology Services, School of Infection, Inflammation and Immunology, College of Medicine and Health, University of Birmingham, Edgbaston, UK; b Emerging Pathogen Serology Group, Vaccine Development Evaluation and Preparedness Centre, UK Health Security Agency, Porton Down, Wiltshire, UK; c School of Chemical Engineering, College of Engineering and Physical Sciences, University of Birmingham, Birmingham, UK

**Keywords:** Mpox, antibody, vaccination, seroepidemiology, infection

## Abstract

Mpox is an ongoing threat to international health security requiring ongoing research. Antibody tests are needed for surveillance studies to understand clinical and sub-clinical transmission risks and respond to outbreaks. With the objective of having a simple and low-cost antibody platform, we developed a laboratory ELISA kit combining four immunodominant mpox proteins to quantify mpox IgG antibody concentration and determine serostatus. The combined assay returns one data point (mpox IgG ratio) per sample. UK serum samples from individuals' post-infection, post-vaccination and those with no known history of mpox infection or vaccination were used to evaluate the assay. The assay effectively discriminated individuals with prior infection or vaccination combined from those without (AUC ·98 [95% CI ·95–1·00], *p* < .0001), returning a sensitivity and specificity of 95% (95% CI 86·71–98·68 and 89·92–97·78%, respectively). Findings highlight the potential of the ELISA as an accurate and practical solution to aid access to mpox immunodiagnostics and should be evaluated further in endemic settings.

The outbreaks of mpox in 2022 and 2024 have proven this virus as a major threat to international health security, demanding ongoing research and development efforts. Since the start of 2022 over 140,000 cases of mpox have been reported from 133 countries, with human-to-human transmission ongoing in Africa and travel associated cases still being reported in various countries [1].

Antibody tests are required at an individual level to confirm serostatus and inform vaccine need, understand clinical and sub-clinical transmission risks, and at a population level to enable surveillance studies to control and respond to outbreaks. This is of heightened importance if vaccine doses are limited and need to be prioritized based on risk. Antibody measurement will also play a key role in evaluating immunogenicity of mpox vaccines under development [2]. As such we need readily available tools that permit comparable antibody detection and quantitation across locations and populations.

A recent study demonstrated poor sensitivity of mpox antibody lateral flow tests and a current lack of acceptable point of care immunodiagnostics [3]. Until suitable community-based tests are developed and verified, serological assessment must be undertaken in a laboratory setting. Several laboratory methods developed for mpox serological assessment have been developed and evaluated [4–7] but real-world application of these have typically been constrained by specialized technology platforms and/or the need to measure multiple antigens, with associated cost, time and expertise. Consequently, these assays are challenging to translate, and sustain, in resource limited settings. We sought to develop a combined laboratory ELISA that could accurately quantify mpox antibody concentration and determine serostatus using the lowest number of mpox antigens possible and a single readout to minimize cost and complexity.

Utilizing data from recently published studies [4,6], we devised a combination of four immunodominant mpox proteins (B2R, B6R, E8L and A27) to enable parallel detection of mpox-specific IgG antibodies derived from both infection and vaccination. Previous data regarding individual antigen performance in relation to vaccination and infection demonstrates proteins B2R, B6R, E8L provide complementary coverage and generate antibody signal across individuals’ post-vaccination and A27 provides detection of infection specific antibodies [4]. These antigens were shown to have high discriminatory potential for infection or vaccination in single antigen ELISAs (area under ROC curve 0.91 to .99, *p* < .001) [4].

The assay was developed into a simple kit format, including the recombinant antigen mix dried into plates and pre-dispensed reagents, to reduce logistical barriers and facilitate shipping and use within different laboratories. The kit comprised of anti-human IgG antibody (conjugated to horseradish peroxidase[HRP]), substrate (TMB), stop solution (sulphuric acid) and sample and wash buffers (PBS and Tween-20). Pre-diluted serum samples (1 in 200 in sample buffer) were added to the plate and incubated for 1 h. After washing, the anti-human IgG-HRP is added and incubated for 1 h. After a final wash step, substrate is added for 15 min followed by stop solution. For each serum sample measured, the assay provides a single readout of optical density (OD, read at 450 nm). In the absence of an international standard with assigned units enabling development of internal calibration material, plate to plate variation in absolute OD was mitigated through adjustment using the National Institute for Biological Standards and Control (NIBSC) working reagent for anti-monkeypox antibodies (22/218) to return an IgG antibody ratio. A 6-point curve of the working reagent is prepared using a 1 in 2 dilution starting at 1 in 100 and the 1 in 800 dilution (located at the seropositivity cut-off) is used as the divisor for sample ODs to provide an IgG mpox antibody ratio.

We used serum samples (N = 187) from three clinical cohorts to evaluate the assay: post-infection (mpox-convalescent 2022 Clade IIb and 2018 Clade II, n = 29), post-vaccination (IMVANEX-vaccinated, n = 33), and negative healthy donors (individuals with no known history of mpox infection or IMVANEX vaccination, n = 125). Inclusion/exclusion criteria for this assay evaluation study were based on mpox history only (known infection, known vaccination, neither [presumed none]).

Samples obtained from IMVANEX Smallpox-vaccinated individuals were obtained through written and informed consent through the UK Heath Security Agency Research and Ethics Committee for assay validation. NHS Research Ethics Committees (REC) granted approval for sampling from previous MPXV-infected individuals under reference 22/HRA/3321, with residual and fully anonymised serum from individuals with prior MPXV infection sourced from diagnostic laboratories for surveillance, assay performance assessment, validation and public health monitoring. Healthy donor serum was used under NHS REC reference 20/HRA/1817 where donors consented for research studies.

Cohort differences in mpox IgG antibody ratio were evaluated using the Kruskal Wallis test with Dunn’s post-hoc test. ROC analysis was performed to evaluate if mpox IgG ratio could distinguish between those with infection and/or vaccination history from those without. Spearman’s rank correlation was used to assess if there was a relationship between time since infection or vaccination and IgG antibody ratio.

The combined four-antigen ELISA detected significantly higher mpox IgG antibodies in both post-infection and post-vaccination cohorts compared with negative donors. The assay effectively discriminated individuals with prior infection or vaccination combined from those without (AUC·98 [95% CI 95–1·00], *p* < .0001), returning a sensitivity and specificity of 95% (95% CI 86·71–98·68 and 89·92–97·78%, respectively) for an IgG ratio cut off of ≥ 1.33. When analysing post-infection and post-vaccination cohorts separately vs negative donors, similar differentiation was found (AUC·96 and·99 [95% CI 89–1.00 and 99–1] respectively, *p* < .0001). The best IgG ratio cut-off for vaccination was the same as both cohorts combined (1.33), with 97% sensitivity (95% CI 84·68–99·84%) and 95% specificity (95% CI 89·92–97·78%). Whereas when trying to distinguish infection only from negatives, a higher IgG ratio cut-off of 1.74 returned the best combination of 93% sensitivity (95% CI 78·04% to 98·77%) and 99% specificity (95% CI 95·61% to 99·96%). Applying the same IgG ratio cut-off of 1.33 to infection only vs negative, sensitivity remained at 93% and specificity 95% (95% CI 78·04% to 98·77% and 89·92% to 97·78%, respectively). There was no significant correlation between days post infection or vaccination and mpox IgG antibody ratio. Data was available for 144/187 samples the MpoxPlex (Luminex) platform [6] and results, in terms of seropositive/negative, were assessed with an overall agreement of 82 % (Figure 1).

**Figure 1.**
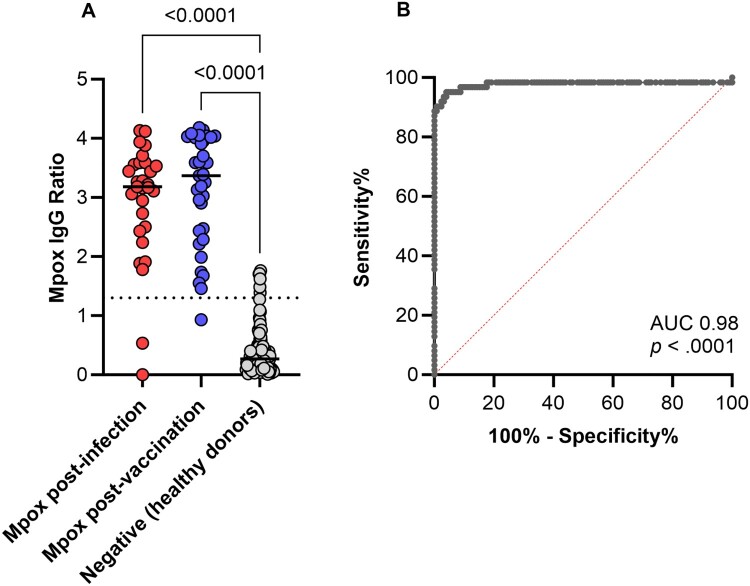
Mpox antibody levels and cohort discrimination using a combined antigen ELISA. Data is shown for mpox post-infection (mpox-convalescent n = 29, 24–113 days post infection), mpox post-vaccination (IMVANEX-vaccinated n = 33: 24–77 days post 1st dose [n = 8], 14–84 days post 2nd dose [n = 24], 14 days post 3rd dose [n = 1]), and negative healthy donors – those with no known history of mpox infection or IMVANEX vaccination, (n = 125) cohorts. For each serum sample, the assay provides a single readout of optical density. This is adjusted using the National Institute for Biological Standards and Control (NIBSC) working reagent for anti-monkeypox antibodies (22/218) to provide an IgG antibody ratio. Panel A shows a statistically significant higher mpox IgG ratio detected in the post-infection and post-vaccination vs negative cohort (p < 0001, Kruskal-Wallis test with Dunn’s post-hoc test). The lines indicate the median of each cohort, and the dotted line indicates a seropositivity cut-off of 1·33. Panel B shows the ROC curve for negative samples vs positive (post-infection and post-vaccination combined) with an AUC of 0·98 (95% CI ·95–1·00) p < .0001. An IgG ratio cut off ≥ 1.33 provided sensitivity of 95·16% (95% CI 86·71–98·68) and specificity of 95·20% (95% CI 89·92–97·78%). The IgG ratio of 1.33 was a ROC analysis-derived cut-off identifying the optimal threshold in this sample and combined infection and vaccination cohort, balancing sensitivity and specificity.

## Discussion

Our findings highlight the need to ensure mpox immunodiagnostics are not only accurate but practical and cost effective for accessibility in a range of laboratory settings. ELISAs are routinely used in clinical laboratories worldwide, allow multiple patient samples to be screened simultaneously, are low cost to manufacture, and can provide reproducible and comparable data between operators and their patient populations. This can overcome the requirement to invest in specialised technology platforms, their ongoing maintenance, and reagent costs. Our approach, using a kit format, avoids the need for sites to procure individual reagents. Manufactured kits, providing sufficient performance studies have been performed, switches the onus from validation to verification, simplifying integration at an individual laboratory level and reducing both time, cost and expertise required to introduce this mpox assay platform versus an in-house or non-standardised method. Laboratories can adapt the ELISA based on the population they serve and circulating virus type, for example selection of an IgG ratio seropositivity threshold/s that provides optimal sensitivity and specificity in local cohorts and intended use (differentiation of antibodies from infection, vaccination or both). The ELISA also provides scope for users to utilize dried blood spot samples if phlebotomy presents logistical or resource barriers. By returning one data point per sample rather than multiple antigens measured independently, a combined ELISA can aid and accelerate interpretation, which is particularly valuable when high-throughput screening is required.

There is a current absence of an international reference sera with assigned units for mpox and an antibody correlate of protection. In this scenario, the use of IgG ratio offers an effective quantitative method to identify past exposure and provide information on transmission, seroconversion to vaccination as an indirect measure of vaccine efficacy, and quantitative temporal observations following natural or vaccine-induced immune responses. The relationship between mpox antibody levels and other immune parameters requires further investigation. Antibody waning or immunosuppression may result in ELISA seronegativity but that does not infer status regarding cellular and recall immune functions.

Through serosurveillance we can track the true spread of disease, even when sub-clinical and asymptomatic, which has been recorded for mpox [8–10]. Robust epidemiological surveillance of mpox, supported by new test development, is vital for improving health policy impact for disease control [11,12]. For example, this data can help inform vaccine planning and prioritization, deployment and evaluation. Is can also help guide implementation of supportive strategies around disease containment and prevention, and asses their effectiveness. The ability to evaluate humoral immunity to mpox at scale, and across the spectrum of geographical locations and variation in host factors, is also needed to urgently address knowledge gaps in the fundamental immune-biology of this virus and how natural and/or vaccine immune seropositivity can change clinical outcomes, disease transmission dynamics and the scale and direction of disease outbreak.

The current method assessment is not without limitations, including modest sample sizes, restricted demographic diversity, the absence of Clade I samples, and heterogenous sampling windows. External studies in mpox endemic locations with sizable relevant infection, vaccination and control cohorts are now required to further assess the methodology and identify the optimal IgG ratio seropositivity cut-off for relevant populations and locations. Samples were collected from a single time point in relatively short time frame post-infection or vaccination. Antibody analysis that spans sampling over a longer time frame or longitudinally is required to provide insights into antibody kinetics, including potential waning.

In addition, the ELISA platform still requires basic laboratory infrastructure. Increasing understanding of both where and who can adopt this laboratory method for mpox surveillance, versus end user need for community point of care tests, would be valuable to ensure efforts and resources into diagnostic development are mapped to requirements.

## Conclusions

Improving capability for mpox antibody measurement remains a critical tool in the repertoire of public health measures that are needed to direct effective countermeasures for disease control. Our ELISA approach offers a promising method for mpox antibody measurement and identification of seropositivity, warranting further evaluation in relevant settings. The combined ELISA is now undergoing evaluation in Rwanda (Mpox CARE NCT06887556) for its suitability for Clade I seroepidemiology and vaccine studies in endemic populations. The ELISA will be validated for serum and dried blood spot samples to facilitate community or remote sampling without the need for venous blood collection.
